# Ultrasonographical Assessment of Caudal Vena Cava Size through Different Views in Healthy Calves: A Pilot Study

**DOI:** 10.3390/vetsci9070308

**Published:** 2022-06-22

**Authors:** Hélène Casalta, Valeria Busoni, Justine Eppe, Sigrid Grulke, Anne-Christine Merveille, Nassim Moula, Kris Gommeren

**Affiliations:** 1 Clinical Department of Production animals, Faculty of Veterinary Medicine, University of Liège, 4000 Liège, Belgium; justine.eppe@uliege.be; 2 Clinical Department of Companions animals, Faculty of Veterinary Medicine, University of Liège, 4000 Liège, Belgium; vbusoni@uliege.be (V.B.); acmerveille@uliege.be (A.-C.M.); kris.gommeren@uliege.be (K.G.); 3 Clinical Department of Equines, Faculty of Veterinary Medicine, University of Liège, 4000 Liège, Belgium; sgrulke@uliege.be; 4 Department of Veterinary Management of Animal Resources, Faculty of Veterinary Medicine, University of Liège, 4000 Liège, Belgium; nassim.moula@uliege.be; 5 GIGA—Animal Facilities—ULiège—B 34, 4000 Liège, Belgium

**Keywords:** point-of-care ultrasound, caudal vena cava, calf, intravascular volume status

## Abstract

**Simple Summary:**

Ultrasonographic measurements of caudal vena cava and aorta are known as reliable tool to assess intravascular volume status in human. The aim of this study was to evaluate the feasibility to assess caudal vena cava with ultrasound in different sites, and to study effect of sex, age, body weight and breed on measurements in healthy calves. A single investigator took measurements of caudal vena cava by ultrasonography in two anatomic sites (subxiphoid and paralumbar site) in 48 calves aged less than 60 days, and then repeated 2,5 months after the first assessment. The study also evaluates the variability between measurements of three observers on five randomly selected calves. The results show that measurement of caudal vena cava by point of care ultrasound was easily obtained on paralumbar site in calves under 4 months of age, and measurements were correlated with the age of the calves. Further studies could compare caudal vena cava measurements in healthy calves to those in calves suffering from diarrhea or surgical disease to see if caudal vena cava point of care ultrasound could complement other shock evaluation parameters such as blood parameters or arterial blood pressure measurements.

**Abstract:**

Ultrasonographic measurements of the caudal vena cava (CVC) and aorta (Ao) are known as reliable tools to assess intravascular volume status in humans. The aim of this study was to evaluate the feasibility of obtaining ultrasonographical measurements of CVC and Ao in two different views, assess intra- and interobserver variability, and study the effect of sex, age, body weight, and breed on measurements in healthy calves. The diameter and area of CVC and Ao were measured by a single investigator in two anatomic sites (subxiphoid and paralumbar window) in 48 calves aged less than 60 days and then repeated 2.5 months after the first assessment. For intra- and interobserver variability assessment, CVC and Ao measurements were repeated by three observers on five randomly selected calves. CVC and Ao measurements were easily obtained in PV and more difficult to obtain in SV. CVC and Ao area in PV showed high repeatability and reproducibility. A positive correlation was highlighted between age and CVC and Ao measurements in both sites. In conclusion, CVC size assessment by point of care ultrasound can be easily performed at a paralumbar site in calves under 4 months of age and could be used to assess intravascular volume status.

## 1. Introduction

Raising calves from birth to one year without excessive mortality is an important and economic challenge for beef and dairy breeders. 

Neonatal diarrhea can lead to severe dehydration and even hypovolemia in sick calves. Abdominal diseases requiring surgical intervention are commonly encountered in young calves and can lead to life-threatening hypotension secondary to hypovolemia or septicemia [1,2]. Proper management of hypovolemic and distributive shock in calves might improve survival rates [3,4]. 

To date, however, the evaluation of intravascular volume status in calves remains challenging. The diagnosis of acute circulatory failure can be based on a combination of clinical, hemodynamic, and biochemical signs, but recent studies in human and animal critical care medicine have demonstrated that ultrasound evaluation of inferior vena cava (IVC) could be a rapid, immediate, and reliable tool to assess intravascular volume status in critically ill patients [5,6,7,8,9].

In human medicine, the ultrasonographic assessment of the IVC diameter and its respiratory variation seems to correlate with central venous pressure in both adults and children [5,10] and may assess changes in volume status earlier than traditional vital signs [5,6,11]. The IVC diameter is a reliable noninvasive tool to assess the effect of fluid administration in hypovolemic patients [7,11,12] and distinguish fluid responders from nonresponders in spontaneously breathing critically-ill patients [13,14]. In a recent study, Kwon et al. (2016) demonstrated that the abdominal aorta (Ao) and IVC cross-sectional area might be a promising index for the assessment of dehydration and may improve the clinical dehydration scale [15]. 

In companion animal medicine, several studies have described the ultrasonographic assessment of the caudal vena cava diameter (CVC_D_) at different locations as an inexpensive, rapid, and noninvasive marker for the assessment of intravascular volume status [9,16,17,18,19,20]. Different sites to perform caudal vena cava (CVC) measurements have been described in different species, and CVC_D_ is often expressed as a ratio to the aortic diameter (Ao_D_) to compensate for variations in body size [16]. To the author’s knowledge the CVC has never been assessed by point of care ultrasound (POCUS) in calves, nor are there any data on the effect of age or body weight in calves on such measurements. One article described the ultrasonography of the spleen, liver, gallbladder, CVC and portal veins in 6 HF calves from birth to 104 days old, but this article focuses on the evolution of organs adjacent to the rumen during ruminal growth and the transition from a milk to a roughage diet [21]. The availability of portable ultrasound devices in rural medicine may change the approach to diagnosis and management of hypovolemia in young calves in everyday practice. The objectives of this study were to: assess the feasibility of obtaining ultrasonographical measurements of CVC and Ao via the right paralumbar (PV) and subxiphoid (SV) view, as previously described in dogs, in awake healthy calves by veterinarians with different levels of experience in POCUS; evaluate intra- and interobserver variability; and to assess whether there were any effects of sex, age, body weight, and breed on the CVC and Ao measurements or the calculated CVC to Ao ratio.

## 2. Materials and Methods

### 2.1. Animals

Forty-eight calves from beef and dairy herds were included in the study. The study was conducted in two sequences: in the first phase, CVC and Ao were assessed in calves aged less than six weeks old and weighing less than one hundred kilos, in order to facilitate animal restraint. From this group, 5 calves were randomly selected to calculate intra- and interobserver variability. 

In the second phase, CVC and Ao were assessed in the same calves two and a half months later. All calves were considered healthy based on a normal physical examination performed just before ultrasound evaluation. The assessment was performed at least one hour after the calves’ milk meal to avoid stress and subsequent health problems secondary to the research project. Chest girth was taken with a weight tape immediately behind the elbow of every calf after clinical examination to estimate body weight. 

The study was approved by the ethical committee of the University of Liège (protocol 2099). 

### 2.2. Point of Care Ultrasound Examination

POCUS was performed by a single investigator who was trained by a small animal specialist in cardiology (ACM) in both parts of the study.

For intra- and interobserver variability evaluation, three different observers realized ultrasound evaluation of CVC and Ao. The first observer (OBS1) was a board-certified imagist, the second observer (OBS2) performed the rest of the study, and the third observer (OBS3) was a veterinarian with little experience in ultrasound. OBS1 and OBS2 were trained to perform the CVC and Ao assessment by the board-certified imagist.

POCUS was performed by using a commercially available ultrasound machine (Mindray^®^ DP50VET) with a microconvex curvilinear probe (5–7 MHz). A curvilinear 5 MHz probe was used on larger animals to obtain proper images at the subxiphoid window.

### 2.3. POCUS Views

**Figure 1:**
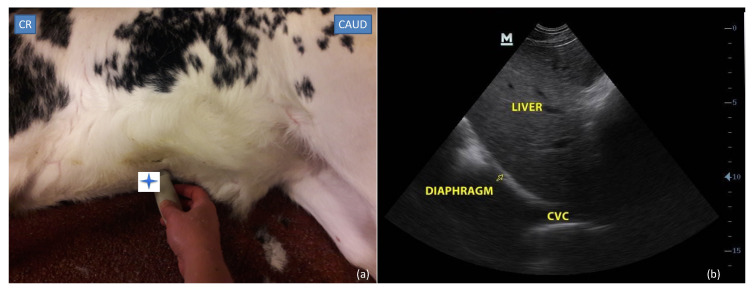
Subxiphoid site: (**a**) probe placement site and (**b**) ultrasonographic view of caudal vena cava (CVC) crossing the diaphragm (Yellow arrow). The blue star shows the position of the probe marker. CR: cranial; CAUD: caudal.

**Figure 2:**
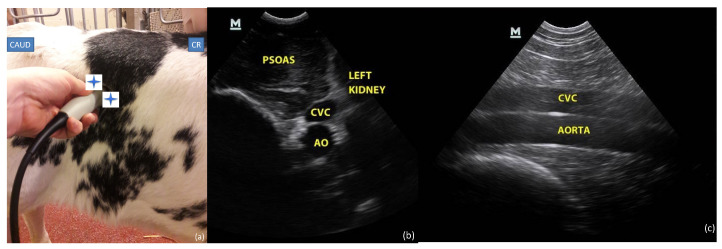
Paralumbar site: (**a**) probe placement site and (**b**) transversal and (**c**) longitudinal ultrasonographic views of caudal vena cava (CVC) and aorta (Ao). The blue star shows the position of the probe marker in transversal and longitudinal view. CR: cranial; CAUD: caudal.

In the first part of the study including intra- and interobserver variability evaluation, two anatomic sites were assessed by the investigator, to obtain three views of the CVC as described below (one view from the subxiphoid window (Figure 1) and two views at the paralumbar window (Figure 2)). 

In the second part of the protocol, performed two and a half months later, only the paralumbar window views were assessed, because of difficulties assessing the subxiphoid view on larger standing animals and the inability to put the calves in lateral recumbency. 

Anatomic sites were clipped, ultrasound gel was used on the probe, and alcohol was used on the site as required to obtain clear images. Care was taken to avoid collapsing the CVC by putting excessive pressure on the ultrasound probe.

#### 2.3.1. Subxiphoid Window

The transducer was placed longitudinally under the subxiphoid process on a standing calf or placed in right lateral recumbency and angled cranially to visualize the diaphragm. The ultrasound probe was inclined from the left to the right of midline until the walls of the CVC could be clearly identified at the level where the CVC crossed the diaphragm. 

#### 2.3.2. Paralumbar Window

The transducer was first placed transversally on the right flank behind the last rib on the standing calf, allowing the visualization of the left kidney. The probe was then slowly inclined from cranial to caudal until the CVC and Ao were identified in a transversal view. The transducer was then rotated 90° to obtain a longitudinal plane in which the CVC and Ao were parallel to each other within the same frame.

### 2.4. Measurements

For the intra- and interobserver variability assessment, every observer recorded three cineloops of 10 s (B-mode images) at each site for each view. 

For the second part of the study, a single investigator recorded one cineloop of 10 s at each site for each view. Cineloops were reviewed offsite a posteriori to perform measurements.

#### 2.4.1. Subxiphoid Window

Measurements of minimal and maximal CVC diameter (CVCmin and CVCmax) were performed at the subxiphoid window. Minimal diameter is supposed to correspond with the end of inspiration when negative intrathoracic pressure is the highest and blood is displaced from the abdominal CVC to the right atrium, whilst maximum diameter corresponds with the expiratory phase when positive thoracic pressure decreases venous return from the CVC to the right atrium. CVCmin and CVCmax were measured by identifying the dorsal and the ventral walls of CVC at the level of the diaphragm.

#### 2.4.2. Paralumbar Transversal and Longitudinal View

As the CVC diameter does not tend to change throughout the respiratory cycle at the paralumbar level, due to the distance from the thoracic cavity, a single measurement of each vessel (CVC and Ao) was made in the paralumbar view. The investigator measured the maximum diameter of the Ao from inner wall to inner wall. Because of the irregular shape of the CVC in a transverse view, the CVC area (CVCa) and the Ao area (Aoa) were assessed, rather than their diameter. The CVC and Ao area index was calculated as the ratio of CVCa and Aoa. 

In the longitudinal view, the maximum diameter of CVC and Ao were taken perpendicularly of the vessel’s walls, from inner wall to inner wall. The CVC and Ao diameter index was calculated as the ratio of CVC and Ao diameters.

### 2.5. Statistics

Data normality was evaluated using the Shapiro Wilk test. Depending on the distribution characteristics, mean and standard deviation (±SD; normally distributed data) or median and interquartile range (IQR; non-normally distributed data) were calculated for all the ultrasound measurements recorded.

For the intra- and interobserver study, a general linear model was used to assess the effect of the observer, repetition or interaction of the observers, and observer repetition of the CVC and Ao measurements on calves. An effect of the observer, the repetition or interaction of the observers, and observer repetition on CVC and Ao measurements was present when *p*-value < 0.05.

Linear regression models were used to investigate and assess correlations between the CVC and Ao measurements and age (in days) and estimated bodyweight (kg) of calves. For each regression, a coefficient of correlation and *p*-value were estimated. 

A general linear model was used to identify the effect of sex, breed, and herd on ultrasound measurements. A *p*-value < 0.05 was considered significant for all statistical calculations. 

All statistical analyses were performed using commercially available software (Xlstat (https://www.xlstat.com, accessed on 20 April 2022, Addinsoft), R (https://www.r-project.org, accessed on 20 April 2022, The R Foundation) and SAS (https://www.sas.com, accessed on 20 April 2022, SAS Institute)).

## 3. Results

### 3.1. Demographic Data

In the first part of the study, forty-eight calves from four different herds were recruited, from February 2020 to February 2021. The breeds were Belgian blue (BB) calves (thirty-two), and Holstein Friesian (HF) (sixteen). The median age and weight of this group were respectively 21 days (range 1–41) and 67 kilos (range 33–98), with twenty-six females and twenty-two males. Five calves from the first part of the study were used for the intra- and interobserver agreement study. 

The second part was conducted on available calves in the same herds 2.5 months after the first assessment. This group includes seventeen BB calves, seven females and ten males, with a median age of 112 days (range 100–126) and a median weight of 134 kilos (range 103–170).

### 3.2. Intra- Interobserver Agreement

For the intra- and interobserver agreement study, ultrasound measurements (Ao diameter, CVC long axis, Ao and CVC area, CVCmin, and CVCmax) and the calculated values (the CVC and Ao diameter and area index) from the paralumbar (longitudinal PV (PV-long) and transversal PV (PV-trans)) and subxiphoid views are reported as mean (±SD) for each observer (OBS1, OBS2 and OBS3) in Table 1, with *p*-values of effects of the observer, the repetition and interaction between observers and repetition on those values. Statistical analysis showed good repeatability and reproducibility for Aod, Aoa, CVCa, and CVCmin (*p*-values > 0.05 meaning there was no significant effect of the observer, repetition or interaction of the observers, or observer repetition on CVC and Ao measurements). Repeatability was also good for all remaining measurements, but interobserver variability was higher for CVCd (*p*-value = 0.03), CVCd and Aod (*p*-value = 0.002), CVCa/Aoa (*p*-value = 0.005), and CVCmax (*p*-value = 0.02) measurements. Nevertheless, reproducibility for all measurements was good when the measurements were repeated (*p*-value > 0.05 for the interaction between observer and repetition).

### 3.3. Point of Care Ultrasound Views and Measurements

#### 3.3.1. Subxiphoid Window

**Figure 3:**
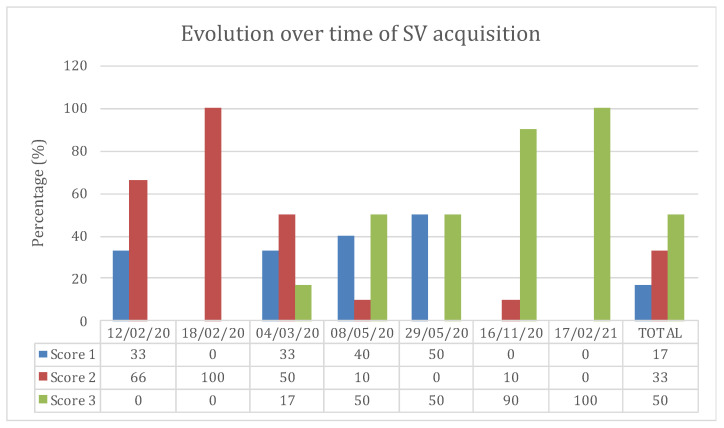
Evolution over time of the percentage (%) of the successful SV cineloop acquisition in calves in the first part of the protocol. Each visit is shown as the completion date (day/month/year). Score 1: No cineloop was recorded because of the impossibility to visualize the CVC on the xyphoid site. Score 2: Cineloop was recorded but the margin of CVC was unclear which made obtaining measurements of the CVC impossible. Score 3: Cineloop was recorded with well-defined margins of the CVC that allowed precise measurements.

In the first part of the study, cineloops were recorded during seven visits of four different herds between February 2020 to February 2021. An attempt was made to record the subxiphoid view in every calf, but cineloops were not recorded in 20% of cases because of poor visualization of the CVC. Scores from 1 to 3 were attributed to each subxiphoid view assessment in each calf. Score 1 corresponded to the absence of a cineloop recorded by the observer. Score 2 corresponded to a recorded cineloop during ultrasound examination, but an inability to discern clear margins of the CVC to allow for a posteriori measurement. Score 3 was attributed to clear images of the CVC allowing for precise measurement. Evolution over time of the percentage (%) of interpretable views realized at the xyphoid site in calves in the first part of the protocol is reported in a graphic (Figure 3). On the first visit, cineloops were not recorded in 33% of the cases (Score 1). In the rest of the calves assessed during this first visit, cineloops were recorded but the CVC was poorly defined, rendering assessment impossible (Score 2). On the last study day, cineloops were recorded in 100% of the calves with clear images of the CVC, allowing precise measurements (Score 3). 

**Figure 4:**
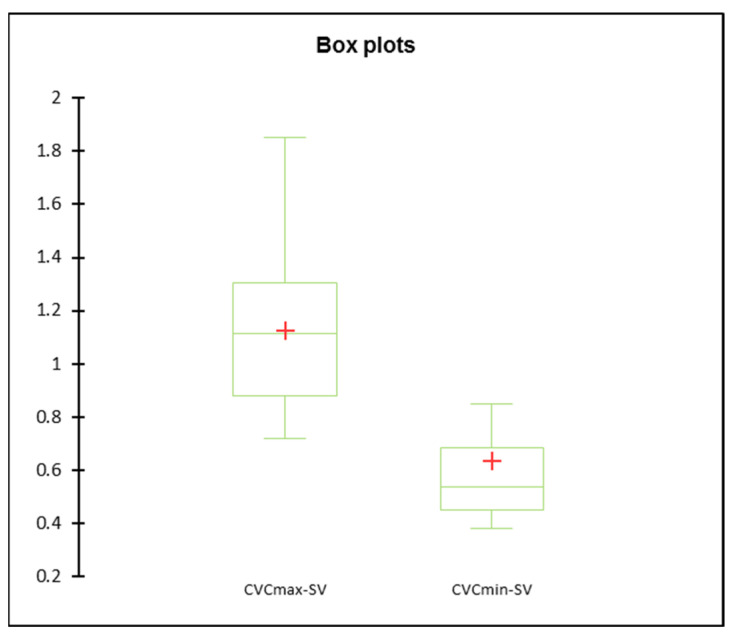
Box and whisker plots of the CVC measurements at the subxiphoid site when the CVC crossed the diaphragm. The CVC_min_ is CVC minimal diameter and the CVC_max_ is CVC maximal diameter measured.

The ultrasound measurements of CVCmax and CVCmin from the subxiphoid view are reported as mean (±SD) or median (IQR), 1.12 (±0.29) and 0.53 (0.45–0.68), respectively. Statistical analysis shows a significant difference between CVCmax and CVCmin with a *p*-value < 0.001 (Figure 4). No correlation was highlighted between CVC measurements at the subxiphoid view and age, weight, breed, sex, or herd. 

In the second part of the study, no cineloops were recorded of the subxiphoid view because this site was difficult to assess in calves weighing over a hundred kilos that were not used to being manipulated.

#### 3.3.2. Paralumbar Transversal and Longitudinal View

In the first part of the study, views from the paralumbar site were recorded on 94% of the calves, and interpretable in 92%. The ultrasound measurements (Ao diameter, CVC long axis, Ao and CVC area) and the calculated values (the CVC and Ao diameter and area index) from the paralumbar views (PV-long and PV-trans) are reported as mean (±SD) or median (IQR) in Table 2. 

**Figure 5:**
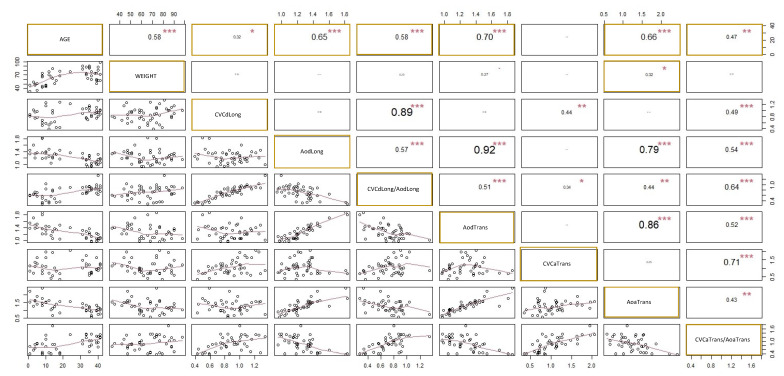
Correlation coefficients (R^2^) between calves’ age and weight and caudal vena cava (CVC) and aorta (Ao) measurements at paralumbar views (PV). ***: *p* < 0.001, **: *p* < 0.01, *: *p* < 0.05. CVCdLong and AodLong are respectively CVC and Ao diameter at longitudinal PV, and CVCdLong and AodLong are the CVC and Ao diameter index in longitudinal PV. AodTrans is Ao diameter in transversal PV, CVCaTrans and AoaTrans are respectively CVC and Ao area at transversal PV, and CVCaTrans and AoaTrans are the CVC and Ao area index in transversal PV.

A high significant linear correlation was found between the age of the calves and CVC and Ao measurements made at paralumbar views (Figure 5) except for the CVC area in transversal PV. A light correlation was found between calves’ weight and Ao area in transversal PV (R^2^ = 0.32, *p* < 0.05). A general linear model analysis showed statistically significant effect of the herd on CVCd (*p* = 0.03), Aod (*p* = 0.005), CVCd and Aod (*p* = 0.004), and Aoa (*p* = 0.002) but no effect of the breed or sex. No effect of the herd, breed or sex was highlighted in CVCmin, CVCmax, CVCa, or CVCa and Aoa (*p* > 0.05). Statistical analysis also showed an effect of the herd on age and weight (*p* < 0.001 for both).

In the second part of the study, which was performed on the same calves but when they were two and a half months older, the paralumbar site was accessed in every calf. Cineloops were recorded and interpretable in 98% of the calves. Ultrasound measurements (Ao diameter, CVC long axis, Ao and CVC area) and the calculated values (the CVC and Ao diameter and area index) from the paralumbar views (PV-long and PV-trans) are reported as mean (±SD) and compared to measurements made in the first part of the study in Table 3. Statistical analysis of paired means shows significant differences between all measurements except for Aoa in PV-trans (Table 3).

## 4. Discussion

Numerous studies have been performed on humans, small animals, and more recently on horses, but our study was the first one to evaluate the feasibility of ultrasonographic CVC assessment through different views in calves. 

In this study, we demonstrated that CVC can be easily assessed via POCUS by trained nonspecialist operators at the right paralumbar site in calves under 4 months old. Moreover, CVC and Ao areas measured in the transversal paralumbar view have high reproducibility and repeatability. The results are comparable with a study published by Darnis et al. (2019) that showed a good inter-rater agreement for CVC diameter measured at the PV in healthy awake dogs [16]. 

However, the results show that reproducibility for CVC diameter measurement in PV-long, both the CVC and Ao diameter and area index, and CVC_max_ in subxiphoid view is lower, and repetition may be needed to overcome differences between observers. Only a few studies in human medicine have investigated intra- and interobserver agreement, but a good interobserver agreement is generally found [5,22]. Del Prete et al. (2021) showed moderate intra- and interobserver agreement for CVC and Ao sonographic measurements, with a lower variability for Ao diameter [19]. 

Our study shows weak interobserver agreement for views performed on the subxiphoid site, this result is in line with the result of a study of interobserver agreement of echographic CVC measurements performed on dogs, where reproducibility of CVC measurements in the subxiphoid view appears to be low [23]. The variations in measuring CVC may occur for several reasons. Although attention is made to putting minimal pressure on the probe when measuring the CVC, there may be differences in the pressure exerted on the probe by the observers that would modify the CVC shape [19,23]. At the subxiphoid view, movements of the CVC occur in both mediolateral and craniocaudal directions during the respiratory cycle, and this movement may be incorrectly interpreted as ICV diameter variation on ultrasound [24]. The CVC could also be measured at a point that is not the true maximum diameter, and Ao sometimes could be mistaken for the CVC because of the location just under the CVC near the diaphragm in the subxiphoid longitudinal view.

The use of the CVC and Ao index shows lower reliability than CVC and Ao measured alone.

Our study shows high repeatability for all CVC and Ao measurements in PV and SV, but the CVC and Ao area measured in the transversal paralumbar view have high reproducibility. Our results are consistent with the study published by Kwon et al. (2016), who show that, because of the elliptic shape of the CVC in transversal PV, measuring the cross-sectional area seems to be more accurate than the maximal diameter [15].

Our study shows differences between CVC_min_ and CVC_max_ in SV. This observation suggests that, as in other species [5,8,10,20], the CVC diameter measured when crossing the diaphragm is influenced by the respiratory cycle in calves. 

We highlighted a positive correlation between age and CVC and Ao diameter and area measured at subxiphoid and right paralumbar sites in calves. Ultrasonography of the CVC visualized between the 9th and the 12th intercostal space in HF calves demonstrated that the circumference of the CVC increases with age [21]. Studies performed on healthy children also showed a positive correlation between age and IVC and Ao diameter, and, inconsistently, with the IVC and Ao index [25,26,27,28]. Del Prete et al. (2021) showed a correlation between age and the long axis of CVC and the CVC and Ao diameter index [19]. In small animals, echocardiographic dimensions seem to increase with increasing body weight rather than age [29], and allometric scaling was used to calculate reference values for CVC measurements according to body weight [16].

In our study, statistical analysis showed an effect of the herd on CVC and Ao measurements in PV-long, but no effect of breed or sex. We can see that age was significantly different between the five herds, and this difference could explain the “herd effect”. Herds who provide calves for this study are clients of the Veterinary Clinic of Ruminant of Liège, and regular interventions and herd visits are performed by our team. During such visits, we could observe some management differences between herds. In one BB herd, our study showed a negative correlation between age and weight (R^2^ = −0.49, *p*-value = 0.04), and this observation could be explained by a proteocaloric malnutrition of calves less than 1 month old discovered during a herd visit. Consequently, despite a normal physical examination at the time of the ultrasound evaluation, calves could have experienced diseases that might have influenced CVC measurement values. In contrast, there was a BB herd with a positive correlation between age and weight (R^2^ = 0.83; *p*-value = 0.0002), and this result correlates well with the good calf growth and management seen in this herd. This disparity between calves of the same breed could also explain the lack of correlation between weight and CVC and Ao measurements in our study, except for one measurement (Ao area in PV-trans).

## 5. Limitations of the Study

This study identified some practical limitations for the POCUS assessment of the CVC in calves, particularly regarding the possible views to assess the CVC. No attempt was made to assess the hepatic view, as it was considered to be one of the most complicated in dogs [16], with limitations of weight and the deep-chest conformity. In one study performed by Braun et al. (2013), the CVC was assessed on HF calves between the 9th and 12th intercostal space, but the shape of the CVC varies at this location from triangular to slightly rounded or oval, and this variation may make measurements difficult [21]. Despite the subxiphoid view being the most frequently assessed in humans, particularly in neonates [5,10,30], this view was not assessed in calves weighing more than 100 kg. The deep chest renders the CVC assessment with a 5 MHz probe difficult as the reachable depth is limited, and it was also difficult to safely access the subxiphoid appendix with the probe in calves under 100 kg that was not used to being manipulated without risk to the observer or the material. Right paravertebral views were easily obtained in 92% of the calves with minimum training, regardless of their age or weight, but sometimes difficulties were encountered in visualizing the CVC and Ao, mostly due to the presence of the colon and gas-filled small intestines. Similarly, in humans, intra-abdominal bowel gas could negatively impact the acquisition of IVC images [31]. 

Generally, in calves of different ages and weights, the subxiphoid view was more difficult to assess than the right paralumbar views, with CVC measurements only accurately performed at this site in 50% of the calves. Most of the time, the CVC could not be visualized because of a distended abomasum pressing against the diaphragm or intra-abdominal bowel gas. Sometimes the power of the probe was not sufficient to access the CVC when the thorax was deeper than 20 cm. This result shows that, contrary to what has been described in other species [18,20], the subxiphoid site could not be used as a reliable tool to ultrasonographically evaluate CVC in calves. However, evolution over time of the percentage (%) of interpretable views realized at the subxiphoid site in calves in the first part of the protocol shows that the technical ability to evaluate CVC at this site could improve with practice (Figure 5).

Three different observers were chosen for the intra- and interobserver agreement, to describe consistency between measurements made by different observers on the ultrasonographically assessed CVC. The board-certified imaging specialist was included in the study as a reference in the CVC POCUS, and her measurements were compared with measurements performed by a resident and rotating intern in the European College of Bovine Health Medicine (ECBHM) and trained by the board-certified imaging specialist. Ideally, the different observers of this interobserver agreement study would have been totally independent and trained separately by different people. However, this is rarely possible in research projects assessing novel techniques. 

In this study, all views were performed either on standing calves or calves in lateral recumbency, and the impact of the calf’s position was not studied. In human medicine, the influence of patient position on inferior vena cava (IVC) diameter differs with studies. Nakao et al. (1987) showed that the IVC diameter and area decrease from the right lateral to the left lateral to the supine position [32]. However, in a study performed on dialysis patients, Panebianco et al. (2014) showed no IVC metrics changes between patients in supine or upright positions. They suggested that IVC was more sensitive to the compressive forces exerted by abdominal organs in right and left rotations than in supine versus upright [33]. In small animals, a pilot study performed on lightly sedated cats indicated that patient position (standing or lateral) has little impact on CVC measurements [34]. Other studies performed on dogs, measured the CVC in left [9,16,17] or in right [35] recumbency, but the impact of recumbency on CVC measurements was not studied. Two studies were performed on healthy foals, one on standing foals with no evaluation of position impact [20], and one on the left and right recumbency [19] in which the left ventral view showed the best reliability and easy visualization of the Ao and CVC cross sections. In ruminants, the left paralumbar view is not accessible because of the attachment of the rumen dorsally in the left side of the abdominal cavity and its progressive development with the calf’s growth. Ultrasonographic evaluation of the rumen showed that the dorsal sac of the rumen can be evaluated in the cranial left flank from birth and in the caudal left flank from 20 days of age [36]. Most healthy calves are easily contained in the standing position, while sick calves presenting dehydration and hypovolemia are more often evaluated in sternal or in lateral recumbency. Further studies could be performed to compare CVC measurements in different positions in calves. 

## 6. Conclusions

In conclusion, we demonstrated that CVC can be easily assessed ultrasonographically by POCUS-trained nonspecialist operators at the right paralumbar site in calves under 4 months of age but was more difficult to assess at the subxiphoid site. Our study shows that the CVC and Ao area measured at the transversal paralumbar view have high repeatability and reproducibility, and the transversal paralumbar view should be preferentially used for assessing CVC in calves. A positive correlation between age and CVC and Ao diameter and the area measured at subxiphoid and right paralumbar sites in calves was demonstrated. Age correlated reference values for calves could allow the use of CVC and Ao measurement to assess intravascular volume status. Further studies could compare CVC and Ao measurements in healthy calves to those in calves suffering from diarrhea or surgical digestive disease to see if CVC and Ao measurements are related to dehydration or state of shock and could complement other shock evaluation parameters such as L-lactates or arterial blood pressure measurements. 

## Figures and Tables

**Table 1:** Ultrasonographic measurements and calculated variables (mean and standard deviation (±SD)) obtained on images from the paralumbar view and subxiphoid view for each observer (OBS1, OBS2 and OBS3) in the intra- and interobserver agreement study, with *p*-values of effects of the observer (OBS), the repetition (REP), and interaction between observer and repetition (OBS/REP) on those values.

POCUS Views		OBS1	OBS2	OBS3	*p*-Value		
					OBS	REP	OBS/REP
**PV-long**	Ao diameter (cm)	1.17±0.03	1.15 ± 0.03	1.21 ± 0.03	0.45	1.00	1.00
	CVC diameter (cm)	1.06 ± 0.08	1.00 ± 0.12	0.94 ± 0.05	0.03	0.99	1.00
	CVC/Ao diameter index	0.91 ± 0.03	0.88 ± 0.03	0.77 ± 0.03	0.002	0.98	0.99
**PV-trans**	Ao area (cm^2^)	0.91 ± 0.03	0.88 ± 0.03	0.77 ± 0.03	0.79	0.87	0.99
	CVC area (cm^2^)	1.08 ± 0.03	1.02 ± 0.03	1.02 ± 0.03	0.41	0.61	0.99
	CVC/Ao area index	0.99 ± 0.04	0.86 ± 0.04	1.02 ± 0.04	0.006	0.98	1.00
**SV**	CVCmin (cm)	0.33 ± 0.02	0.39 ± 0.02	0.37 ± 0.02	0.09	0.29	0.92
	CVCmax (cm)	0.74 ± 0.04	0.89 ± 0.04	0.85 ± 0.04	0.02	0.99	0.99

PV-long: longitudinal paralumbar view; PV-trans: transversal paralumbar view; SV: subxiphoid view.

**Table 2:** Ultrasonographic measurements and calculated variables (mean and standard deviation (±SD) or median and interquartile range (IQR)) obtained from images of the paralumbar view in 48 calves on the first part of the study.

POCUS Views	Ao Diameter (cm)	CVC Diameter (cm)	CVC/Ao Diameter Index	Ao Area (cm^2^)	CVC Area (cm^2^)	CVC/Ao Area Index
**PV-long**	1.20 (1.09–1.35)	0.86 ± 0.23	0.71 ± 0.22	-	-	-
**PV-trans**	1.25 (1.08–1.39)	-	-	1.26 ± 0.39	1.06 ± 0.42	0.89 ± 0.34

PV-long: longitudinal paralumbar view; PV-trans: transversal paralumbar view.

**Table 3:** Ultrasonographic measurements and calculated variables (mean ± SD) obtained on images from the paralumbar view in 17 calves for the first part of the study (T1) and the second part of the study (T2).

	POCUS View	T1	T2	*p*-Value
**PV-long**	Ao diameter (cm)	1.09 ± 0.10	1.24 ± 0.14	0.0004
	CVC diameter (cm)	0.92 ± 0.14	1.25 ± 0.24	0.00005
	CVC/Ao diameter index	0.84 ± 0.09	0.97 ± 0.19	0.009
**PV-trans**	Ao diameter (cm)	1.13 ± 0.12	1.28 ± 0.13	0.00001
	Ao area (cm^2^)	0.99 ± 0.23	1.05 ± 0.3	0.3269
	CVC area (cm^2^)	1.04 ± 0.35	1.55 ± 0.6	0.002
	CVC/Ao area index	1.05 ± 0.26	0.97 ± 0.19	0.002

PV-long = longitudinal paralumbar view, PV-trans = transversal paralumbar view, Ao = aorta, CVC = caudal vena cava. *p*-value < 0.01 indicates significant difference between measurements in T1 and T2.

## Data Availability

Not applicable.
